# OncomiRs as noncoding RNAs having functions in cancer: Their role in immune suppression and clinical implications

**DOI:** 10.3389/fimmu.2022.913951

**Published:** 2022-09-16

**Authors:** Khalid Otmani, Redouane Rouas, Philippe Lewalle

**Affiliations:** ^1^ Experimental Hematology Laboratory, Hematology Department, Jules Bordet Institute, Brussels, Belgium; ^2^ Hematology Department, Université libre de Bruxelles, Brussels, Belgium; ^3^ Hematological Cell Therapy Unit, Hematology Department, Jules Bordet Institute, Brussels, Belgium

**Keywords:** miRNAs, tumor immune microenvironment, extracellular vesicles, cancer immune modulation, oncomiRs

## Abstract

Currently, microRNAs have been established as central players in tumorigenesis, but above all, they have opened an important door for our understanding of immune and tumor cell communication. This dialog is largely due to onco-miR transfer from tumor cells to cells of the tumor microenvironment by exosome. This review outlines recent advances regarding the role of oncomiRs in enhancing cancer and how they modulate the cancer-related immune response in the tumor immune microenvironment.

MicroRNAs (miRNAs) are a type of noncoding RNA that are important posttranscriptional regulators of messenger RNA (mRNA) translation into proteins. By regulating gene expression, miRNAs enhance or inhibit cancer development and participate in several cancer biological processes, including proliferation, invasion metastasis, angiogenesis, chemoresistance and immune escape. Consistent with their widespread effects, miRNAs have been categorized as oncogenes (oncomiRs) or tumor suppressor (TS) miRNAs. MiRNAs that promote tumor growth, called oncomiRs, inhibit messenger RNAs of TS genes and are therefore overexpressed in cancer. In contrast, TS miRNAs inhibit oncogene messenger RNAs and are therefore underexpressed in cancer. Endogenous miRNAs regulate different cellular pathways in all cell types. Therefore, they are not only key modulators in cancer cells but also in the cells constituting their microenvironments. Recently, it was shown that miRNAs are also involved in intercellular communication. Indeed, miRNAs can be transferred from one cell type to another where they regulate targeted gene expression. The primary carriers for the transfer of miRNAs from one cell to another are exosomes. Exosomes are currently considered the primary carriers for communication between the tumor and its surrounding stromal cells to support cancer progression and drive immune suppression. Exosome and miRNAs are seen by many as a hope for developing a new class of targeted therapy. This review outlines recent advances in understanding the role of oncomiRs in enhancing cancer and how they promote its aggressive characteristics and deeply discusses the role of oncomiRs in suppressing the anticancer immune response in its microenvironment. Additionally, further understanding the mechanism of oncomiR-related immune suppression will facilitate the use of miRNAs as biomarkers for impaired antitumor immune function, making them ideal immunotherapy targets.

## 1 Introduction

Altered regulation of signaling pathways primarily linked to dysregulation of their epigenetic machinery and genetic alterations are major hallmarks of cancer. Tumor behaviors should, however, not be analyzed apart from their microenvironment interactions, which support tumor growth and immune evasion (1). The processes by which cancer cells evade the immune response are numerous, including resistance to cell death (particularly by increasing the synthesis of anti-apoptotic proteins and decreasing pro-apoptotic proteins), producing immunosuppressive molecules, inhibiting the antitumor T-cell and natural killer (NK) responses and the full maturation of dendritic cells and promoting the induction of Tregs, myeloid-derived suppressor cells (MDSCs) and tolerogenic dendritic cells (2). Tumor cells also induce T lymphocyte apoptosis and decrease their immunogenicity. In particular, they modulate immune checkpoint protein expression to silence the immune response to tumor-associated antigens (TAAs). Dysregulated expression of immune checkpoint molecules in different cancers is correlated with increased exhaustion of antitumoral effector lymphocytes (3) and has been considered a major barrier to effective immunotherapy (4). Many studies on cancer development have emphasized the critical role of the tumor microenvironment (TME) in tumor progression. Understanding the interplaying molecular mechanisms in the TME has been a major goal of many research studies to allow reprograming of the TME to drive anticancer responses and eliminate immune suppression (5).

Small noncoding RNAs known as miRNAs are posttranscriptional regulators of gene expression. MiRNAs inhibit expression of their target genes by silencing messenger RNA (mRNA) translation and degrading mRNA transcripts. Consistent with their wide-ranging effects in cancer, miRNAs have been divided into two categories: oncomiRs, which are oncogenic, and TS miRNAs. OncomiRs can undergo gain-of-function in cancer, and TS miRNAs can undergo loss-of-function in tumors. Moreover, several miRNAs exert dual effects, acting as either TSs or as oncomiRs depending on the tumor cell type. This has been demonstrated in various miRNA family including as known tumoral activator miR-30 and miR-21. MiR-30 in pancreatic cancer acts as an oncomiR. The high expression of miR-30 in pancreatic cancer modulated by CD133 promotes migration and invasion of pancreatic cancer cell (6). In glioblastoma, miR-21-3p act as tumor promoter by targeting HNRPK and TAp63 as an important components of the p53, transforming growth factor TGF-β and mitochondrial apoptosis tumor suppressive pathways (7). In human hepatocellular cancer miR-21 promote cell proliferation, migration and invasion by targeting PTEN (8).

The most fascinating aspect of miRNAs is that they not only regulate gene expression in the cancer cells themselves but are also used by the tumor to modify its microenvironment. MiRNAs can be released outside the cell and picked up by nearby cells in which they will also regulate gene expression. MiRNAs are at the center of an intercellular communication network, controlling dynamic multidirectional crosstalk not only between cancer cells and their microenvironment but also inducing cascading effects between the different components of the tumor microenvironment. Immunity-related miRNAs have received much attention, and among other effects, the regulation of immune checkpoint expression by miRNAs in various cancer types has been extensively reported (9).

To communicate with their surrounding microenvironment and to reprogram the functions of various cells, including immune cells, tumors produce exosome (10). exosome serve as a major delivery system to propagate signals to local or distant cells in the TME (11). Exosomes produced in the TME have dual capacity; they can stimulate the function of immune effector cells or impair their antitumor activities. The extracellular tumor microenvironment contains a set of miRNAs from tumor cells and cells of the microenvironment, particularly immune cells. During the early phase of tumor development, miRNAs primarily reflect the antitumor immune response. During later phases, the balance is in favor of oncomiRs produced by the tumor and miRNAs favoring immunosuppression (9, 12).

This review systematically summarizes the role of miRNAs with oncogenic functions that not only contribute to the promotion of tumorigenesis but are also key players in immune cell function regulation of the tumoral surroundings and stresses the significant role of oncomiRs as potential clinical biomarkers and targets for immunotherapies.

## 2 OncomiRs inside cancer cells

### 2.1 How oncomiRs contribute to tumorigenesis

**Figure 1:**
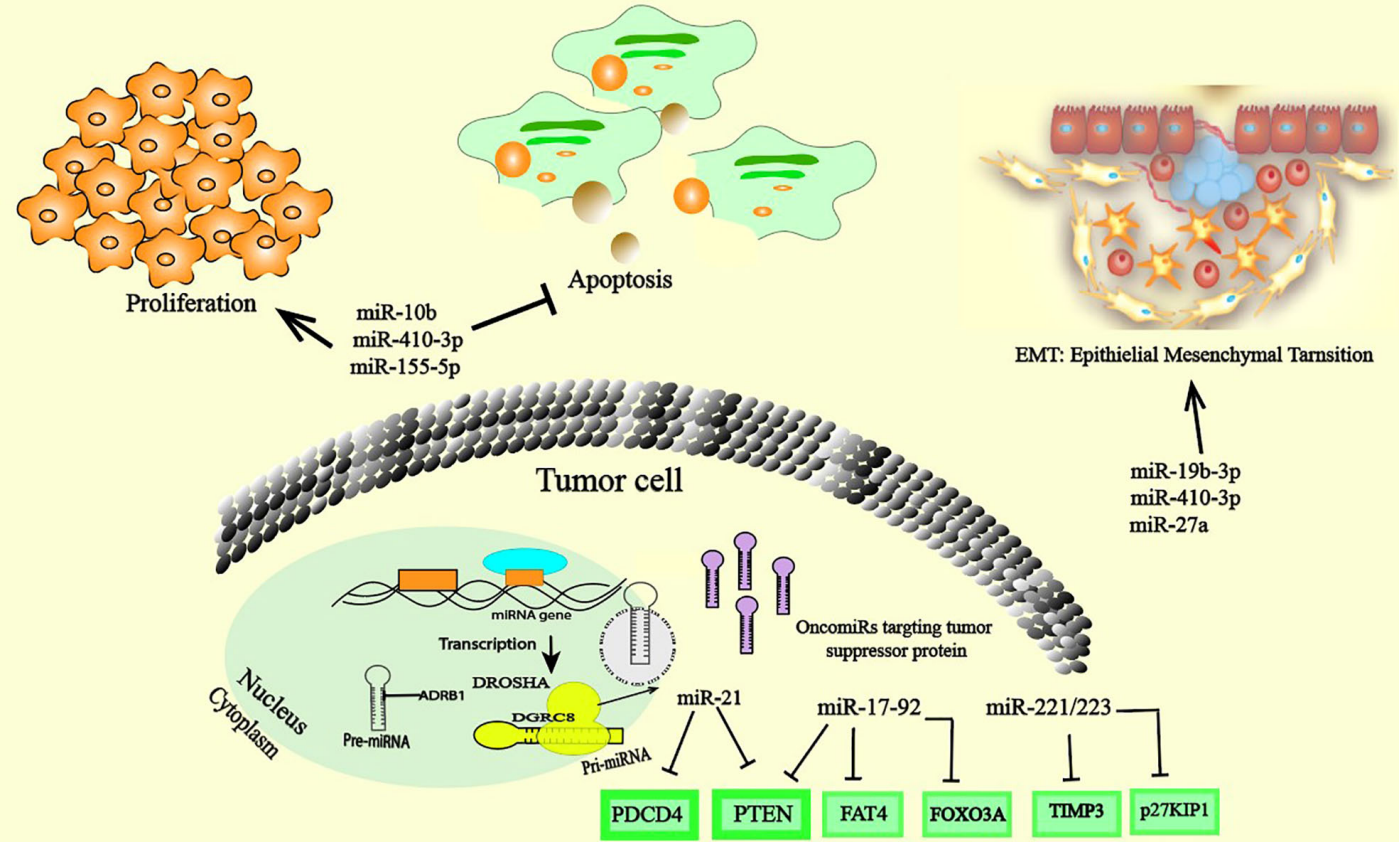
OncomiRs inside cancer cells. The overexpression of oncomiRs in cancer cells promote cancer through the regulation of the expression of multiple tumor suppressor gene targets with cancer-related cellular processes and/or genes that control cell differentiation, proliferation. OncomiRs can also enhance the EMT process, cell migration and invasion.

Cancer cells are characterized by alterations in the expression of multiple genes involved in cell proliferation, survival and death. Changes in the expression levels of these genes are linked to changes in the expression and dysfunction of various regulators, including miRNAs. Currently, many studies have demonstrated the central role of oncomiRs in promoting cancer development through regulating the expression of multiple TS genes involved in cell differentiation and in cell death pathways such as apoptosis. These studies have shown that oncomiRs are significantly upregulated in cancer cells and in the TME, and their increased levels largely contribute to cancer progression (**Figure 1**). However, to date, only a few of these miRNAs have been well characterized. MiR-17–92, miR-21, miR-155, and miR-10b are an important examples of oncomiRs that have received substantial attention. MiR-17-92 is significantly increased in many several cancers, including breast cancer (BC) (13), ovarian cancer (OC) (14), and lung cancer (LC) (15). Recently, a meta-analysis of the role of miRNAs in gene expression regulation identified cancer-associated signatures that underline the high expression of miR-17-5p, which extensively regulates the expression of TS proteins such as Phosphatase And Tensin Homolog (PTEN), FAT Atypical Cadherin 4 (FAT4) and cyclin-dependent kinase 12 (CDK12), demonstrating miRNA-mediated gene expression regulation across tumor types and revealing a major alternative method of gene suppression in addition to mutation, methylation and copy number changes (16). It’s seems that the combination of suppressing many TS gens is responsible for the increase of proliferative capacity and anti-apoptotic effect (17). The suppression of the pro-apoptotic protein Bcl-2-like protein 11 (Bcl-2L12) by miR-17-92 in B cell lymphoma provide a mechanism of apoptosis resistance, and this mechanism probably contributed to lymphoproliferative (18). In addition, a number of studies have highlighted the pivotal regulatory roles of miR-21 and miR-17-92 in apoptotic signaling pathways and proliferation by downregulate the expression of many distinct proapoptotic and antiproliferative targets in various cancer (19) (20). MiR-221/223 are two additional examples of oncogenic miRNAs that are highly expressed during breast tumorigenesis and metastasis. Upon expression, they suppress multiple TS genes, including Cyclin-dependent kinase inhibitor 1B (p27KIP1), Transcription Factor Forkhead box protein O3 (FOXO3A), PTEN, and TIMP Metallopeptidase Inhibitor 3 (TIMP3) (21). Moreover, miR-221/222 was shown to have multifunctional roles in the promotion of epithelial-mesenchymal transition (EMT) in BC by suppressing Notch Receptor 3 (Notch3), ERα, and E-cadherin-induced EMT (21). MiR-21, described as an oncomiR, exhibits high expression in numerous cancer cell types. Likewise, dysregulation of miR-21 expression in cancer has been shown to be associated with the deregulation of many TS gene targets, such as programmed cell death 4 (PDCD4), PTEN (22–25) and FOXO1 (26). Moreover, suppression of miR-21 expression strongly correlates with inhibition of cancer progression. A recent study using miR-21 knockout mice reveals 21 genes as potential targets of miR-21, mostly tumor-associated genes (27). miR-21 knockout leads to extensive upregulation of TS genes and downregulation of oncogenes which indirectly impacts the expression of a network of 310 proteins mainly related to cancer development (27). In clear cell renal cell carcinoma (ccRCC), overexpression of miR-153-5p is related to poor prognosis and promotes tumor growth and invasion *via* inhibition of argonaute 1 (AGO1) (24). Several experiments and clinical analyses suggest that oncomiR function promotes tumor development by negatively inhibiting TS genes involved in apoptosis and regulating proliferation (28). Aberrant expression of miR-10b has been observed in several cancers. Increased expression of miR-10b in CNE1 cells promotes proliferation, migration and EMT in NP69 cells by downregulating epithelial cell markers (E-cadherin and β-catenin) and upregulating the expression of mesenchymal cell markers (fibronectin, N-cadherin, vimentin and Matrix Metallopeptidase 9 (MMP9)) in nasopharyngeal carcinoma cells (29, 30). MiR-410-3p is another oncomiR that displays high expression in several cancers. In prostate cancer, upregulation of miR-410-3p and miR-182-5p are correlated with poor prognosis, and target EMT-associated pathways. miR-410-3p silencing suppresses proliferation and migration through the PTEN/AKT/mTOR axis (17, 31). A recent study in colorectal cancer (CRC) cells also demonstrated that high expression of miR-410-3p activates migration, metastasis and promotes EMT by suppressing ZCCHC10-mediated nuclear factor-kappa B (NF-κB) activation (32). Moreover, miR-19b-3p is an oncomiR reported to promote cancer in several studies. Functional assays showed that silencing miR-19b-3p through circSMARCA5 inhibited the proliferation and migration of NSCLC cell lines by regulating homeobox A9 expression (HOXA9) (33). Furthermore, miR-19b-3p is highly expressed in intrahepatic cholangiocarcinoma (ICC) tissues. Further study revealed that miR-19b-3p directly targets the expression of coiled-coil domain-containing 6 (CCDC6). Overexpression of miR-27a in ovarian cancer activated migration, invasion and induced EMT by directly targeting FOXO1 expression and the Wnt pathway. In contrast, silencing miR-27a induced the opposite effect. MiR-155 has attracted increased attention in cancer and is considered of major importance in cancer development due to its involvement in signaling pathways extensively involved in the immune system and inflammatory modulation (34). The oncogenic potential of miR-155 has been demonstrated in several cancers. miR-155-5p was shown to promote proliferation and inhibiting apoptosis in many cancer type (35). Recently, high expression of miR-155-5p was detected in renal cancer (35). Abrogated expression of miR-155-5p inhibited cancer development and progression. In contrast, in renal cell carcinoma, overexpression of miR-155-5p favors cell proliferation, colony formation, migration, and metastasis by regulating N-cadherin and Zinc finger protein SNAI1 (Snail) (35), associating increased expression with invasive phenotypes and metastasis **(** **Figure 1** **).** Tumor developed a unique metabolic pattern involved in all of its invasive behaviors. Therefore, miRNAs also regulated tumor growth by impacting on their metabolism pathways. As example, in CRC, miR-101-3p was shown to target homeodomain-interacting protein kinase 3 (HIPK3) axis involved in cell metabolism regulation. HIPK3 inhibition by miR-101-3p strengthens oncogenicity by improving aerobic glycolysis (36). Lactate production by glycolysis promotes the acidification of the tumor microenvironment which in turn dysregulated numerous gene expressions in favor of tumor growth.

### 2.2 Mechanism of cancer cell oncomiR regulation

MiRNA expression is controlled at multiple levels, including transcriptional and posttranscriptional regulation (37). Total miRNA expression is globally downregulated in cancer cells and the tumor microenvironment (38); nevertheless, several specific oncomiRs with protumoral action are upregulated. Many mechanisms of oncomiR upregulation exist even if the underlying players are not yet well known. Many proinflammatory cytokines, such as Tumor Necrosis Factor (TNF-α) and interleukins, have been shown to promote the initiation of tumorigenesis in various cancers through aberrant expression of miRNAs. MiRNA expression in cancer is linked to genetic and epigenetic control of the miRNA biogenesis machinery. MiRNA synthesis is usually dysregulated by genetic deletion or amplification and epigenetic methylation of miRNA genomic loci (39, 40). Transcription factor-mediated regulation also often impacts primary miRNA expression. Interestingly, transcriptional mechanisms are most commonly responsible for the upregulation of oncomiRs in cancer cells. The oncogenic transcription factor (c-Myc) was reported to induce the expression of several oncomiRs, such as miR-17-92, miR-21, and miR-10b (41), and its dysregulated expression or function is a common abnormality observed in many human malignancies (42). Moreover, c-Myc downregulates the expression of TS miRNAs (miRNAs), such as the miR-34, Let-7 and miR-15a/16-1 families, in cancer (43). The Telomeric Repeat binding Factor 2 (TRF2) regulated by the Wnt/β-catenin pathway is overexpressed in several human cancer where its promote tumor immune escape and angiogenesis favoring tumor growth and metastasis. TRF2 as a transcriptional regulator of miRNA expression promotes tumor growth, among other things, by regulates miRNAs expression including miR-193b-3p. In CRC, as a transcriptional regulator of miRNA expression TRF2 cooperates with CTCF, a chromatin organization factor, to promote miR-193b-3p expression that inhibits the onco-suppressive methyltransferase SUV39H1 (44). Furthermore, a nontranscriptional mechanism for oncomiR upregulation, implying gene amplification, has been suggested to be responsible for the increased levels of oncomiRs in cancer. For example, in BC, the miR-21 genomic locus was found to be amplified, providing a nontranscriptional mechanism for increased miR-21 expression. Moreover, some of the mechanisms that regulate cancer cell miRNA production are linked to the TME, and hypoxia, acidosis and inflammation are the primary mechanisms that increase cancer cell oncomiR expression. However, the alteration of miRNA profile expression is not only the consequence of dysregulation in signaling pathways but also from more general events, such as limited oxygen supply and acidity (45). Hypoxia is typically present within the TME, and many hypoxia-regulated miRNAs (HRMs), such miR-29, miR-372/373, miR-297, miR-155, miR-21, and miR-210, that are overexpressed in various cancers behave as oncomiRs (46, 47). Hypoxia inducible factor 1 (HIF1) is a key regulator of the expression of numerous miRNAs. HIF1 plays an important role under hypoxic conditions in transcription factors targeting miRNA expression and in direct binding to hypoxia-responsive elements (45). Acidosis is another mechanism that induces increased expression of oncomiRs in the TME. The acidic microenvironment promotes the release of exosome-engaged miRNAs, such as miR-10b, to promote the proliferation of cancer cells in hepatocellular carcinoma (48).

## 3 Extravesicular oncomiRs

### 3.1 Impact of exosomal miRNAs on the TME

The tumor microenvironment is a complex set of cellular and extracellular elements that is in constant interaction with tumor cells. The cellular elements consist of tumor stroma cells (cancer associated fibroblasts (CAFs), mesenchymal stromal cells, endothelial cells) innate and adaptive immune cells, such as T cells, NK cells, and macrophages. The extracellular environment is composed of the extracellular matrix and is rich in active molecules such as growth factors, proteolytic enzymes and cytokines (1). During its development, the tumor both directly and indirectly impacts its microenvironment to make it favorable to its growth and dispersal (1). Many factors, such as cytokines, chemokines, and metabolites, directly produced by tumor cells or indirectly produced by the TME under tumoral influence contribute to tumor growth support and anticancer immune response suppression. Cells of the TME are functionally programmed to favor the tumor. For example, T cells primarily because exhausted and undergo apoptosis or acquire a suppressive immunoregulatory phenotype (Treg cells, Th2 phenotype), and the innate immune compartment is enriched in NK2 cells, MSC2 cells, MDSCs and tumor-associated macrophages (TAMs) (49, 50). Altogether, both cellular and noncellular elements of the TME create a specific niche favorable to tumor cell growth. It is now well established that tumor development and progression are the direct result of the close interaction between cancer cells and their associated microenvironment. During cancer development and progression, a complex molecular interaction occurs between tumoral cells and TME cells. Several miRNAs have been shown to play an important role in this crosstalk, making them key players in determining the contexture of the TME (51, 52). Increased levels of oncomiRs play a critical role in anticancer response suppression by targeting key TS genes and inhibiting the recruitment and activation of effector immune cells. One of these effects is modulation of the expression of immune checkpoint proteins to silence the host immune response to cancer-specific antigens.

To communicate with their surrounding microenvironment and to reprogram the functions of various cells, tumors produce small exosome called tumor-derived exosomes (TEXs) to build a communication network (6). The concept that only miRNAs contained in membrane vesicles are released into the microenvironment is, unfortunately, too reductive. MiRNAs can indeed be cargoes of exosome, but in fact, more than 90% of extracellular miRNAs are not contained in exosome where it is released to environment in association with proteins of the AGO family or HDL lipoproteins, which protect them from degradation by RNase. They can also be carried by apoptotic bodies. vesicles (EVs) are typically divided into three categories based on their size (53). True exosomes are the smallest vesicles, with a size of 30-100 nm (7). Furthermore, Exosomes not only carry miRNAs but also proteins, mRNAs, DNA, and long noncoding RNAs, which are also involved in the regulation of cancer growth (7). The relative importance role of circulating miRNAs has not yet been fully elucidated and is the subject of numerous studies. Exosomes produced in the TME have a dual capacity: they can stimulate the function of immune effector cells or impair their antitumor activities.

Exosomal miRNAs constitute one of the most dynamic facets of the TME collaborative network (40). A major difficulty in this field is the heterogeneity of the contents of EVs experimentally isolated from different patients or from different tissue culture batches, making the validation of results and their generalization complex.

The reprogramming of macrophages by tumor-educated macrophages through exosome carrying oncomiRs leads to the creation of a microenvironment for tumor growth. M2 macrophages in turn take responsibility for cancer cells by releasing exosomes that promote tumor growth and metastasis. M2 macrophage-derived exosomes can promote the progression of colon cancer (41) and gastric cancer (42). Moreover, protected by a carrier, miRNAs can exert their regulatory function beyond their cells of origin. Indeed, circulating miRNAs can be detected in the blood of patients. MiRNAs can regulate gene expression in recipient cells by binding to their target mRNAs (54). Cancer-released exosomal miR-9 and miR-181a transferred to MDSCs suppress the development of early-stage MDSCs by targeting Suppressor of cytokine signaling (SOCS3) and Protein Inhibitor Of Activated STAT Protein (PIAS3) (44). It has also been reported that during cancer progression, cancer cells can utilize exosome-mediated secretion of TS miRNAs to discard miRNAs that suppress several steps of the tumorigenesis process, such as angiogenesis, invasion, anoikis resistance, and lung colonization (55). For example, miR-23b, miR-92, and miR-224 are TS miRNAs found in exosomes released from bladder carcinoma cells (55, 56).

### 3.2 OncomiRs impair the anticancer ability of the immune system

Cancer cells develop multiple mechanisms to counteract the immune system antitumoral attack. A major one is the alteration of miRNA expression both in tumor cells and their microenvironment. Tumor cells aberrantly express several miRNAs, and this deregulation of the expression is characterized by high expression levels of oncomiRs and downregulation of TS miRNAs. In cancer cells, miRNAs can downregulate antigen presentation to impair recognition by T cells. To suppress an effective immune response, cancer cells can downregulate TS miRNAs that inhibit immunosuppressive factors and inhibitory costimulatory molecule expression to directly suppress T-cell and NK-cell activation. MiRNAs also regulate different stromal cells and indirectly impact the recruitment and differentiation of suppressive immune cells, such as Tregs, TAMs and others that are present in the TME (57). A previous study demonstrated that tumor-secreted miR-21 and miR-29a in exosomes in a mouse model of lung cancer, are delivered to immune cells and bind as ligands to receptors of the Toll-like receptor (TLR) family, murine TLR7 and human TLR8. This binding triggers a TLR-mediated prometastatic inflammatory response by activating the NF-κB pathway, which initiates an inflammatory reaction mediated by TNF-α and IL-6, ultimately leading to tumor progression and metastasis (25).

#### 3.2.1 OncomiR suppression of T-cell cytotoxicity

Tumor infiltration by leukocytes is usually considered a good prognostic marker and is necessary for successful immune elimination. Impaired antitumor activities of T cells are a major goal of cancer cells. The growth and development of tumors is often associated with deregulation of antigen expression, which impairs the recognition of tumor cells by immune effector cells (58). This altered antigenic pattern is primarily under the control of several miRNAs. MiR-222 and miR-339 inhibit intracellular cell adhesion molecule 1 (ICAM-1) expression on the surface of cancer cells and contribute to the impairment of cytotoxic T lymphocyte (CTL)-mediated tumor cell lysis (59). The interaction of ICAM-1 with lymphocyte function-associated antigen 1 (LFA-1) has been reported to be important for potent activation of T-cell cytotoxicity (60). Moreover, miR-9, which is overexpressed in many cancers, was shown to downregulate the expression of MHC class I, hindering the recognition of malignant cells by the antitumor immune response (61). Exosomes carrying miR-212-3p derived from pancreatic tumor cells (PANC-1) impair the functions of dendritic cells, which decrease MHC II expression, inducing immune tolerance (27).

Frequently, cancer cells escape lysis by cytotoxic T lymphocytes (CTLs) and NK cells by downregulating human leukocyte antigen (HLA) class I expression on their surface (62). T cells can potentially recognize tumor cells and eliminate them. Furthermore, tumor cells have developed multiple mechanisms to convert T cells from cytotoxic effectors to suppressors, characterized by downregulated expression of costimulatory molecules such as CD27 and CD28 (63). Importantly, many studies indicate that exosomal miRNAs regulate T-cell activity through direct binding and fusion or indirectly by changing the phenotypes of other immune cells, such as macrophages and DCs, reprogramming them toward a protumoral profile (64). However, the effect of miRNAs transferred from tumor-derived exosome to immune cells is a central platform based on miRNA-induced alteration of key signaling pathways in immune cell activity to initiate immunosuppression. In CRC, miR-146a was found at high levels in circulating exosome, which promoted tumorigenesis and decreased tumor-infiltrating CD8+ T cells (65). MiR-146 was found to be an important negative regulator of innate and adaptive immune system activation and oncogenic transformation in CC (66). This is in line with a previous study showing that hepatocellular carcinoma (HCC)-derived exosome containing miR-146a enhance M2 macrophage polarization and consequently promote T-cell exhaustion by increasing Programmed cell Death protein 1 (PD-1), T cell immunoreceptor with Ig and ITIM domains (TIGIT), and cytotoxic T-lymphocyte antigen 4 (CTLA4) expression (67) and suppress CD8+ T-cell activity by enhancing TIM-1+ Breg cells, consequently accelerating tumor development (68).

**Figure 2:**
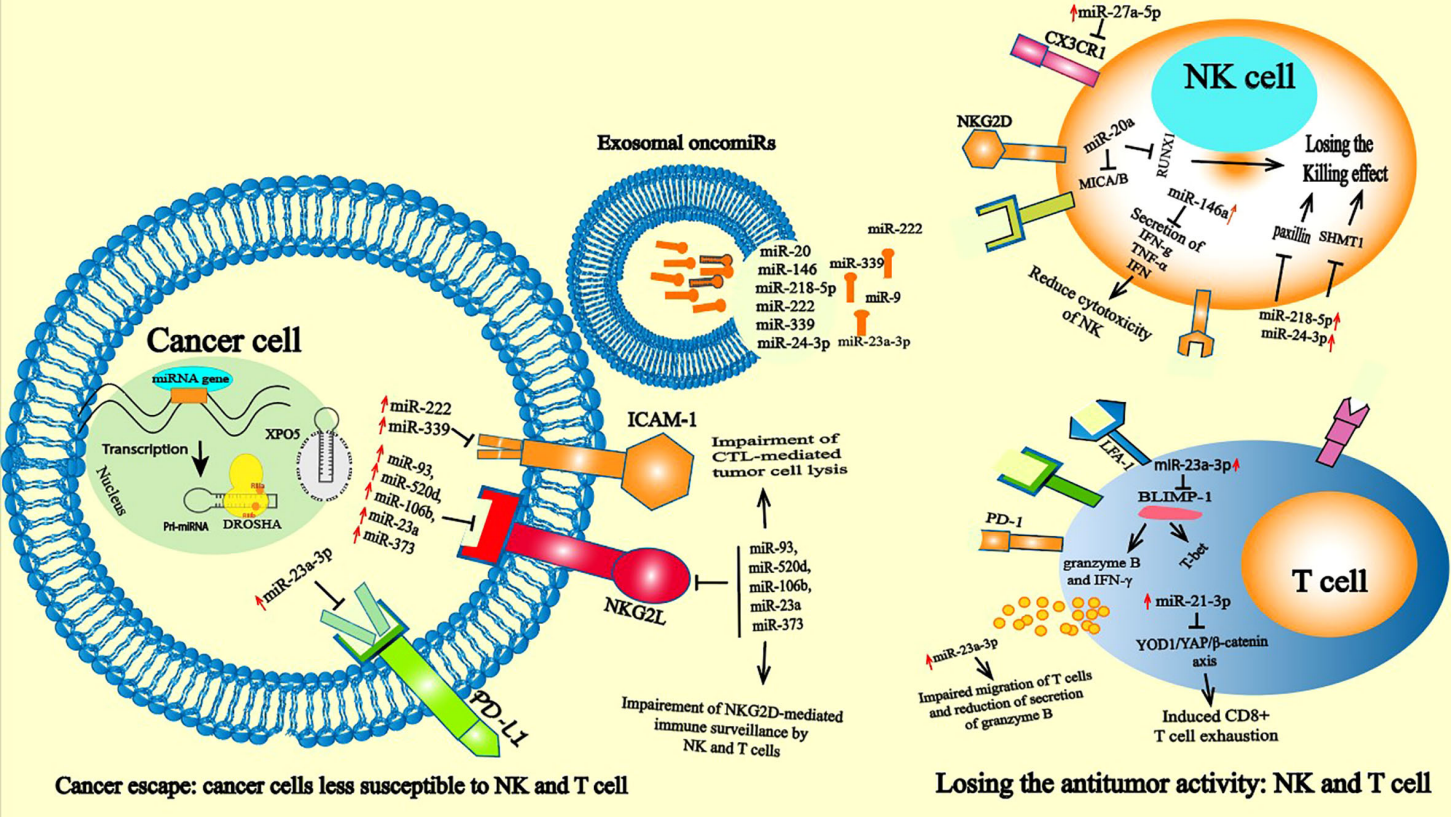
oncomiRs induced immune suppression: Leukocyte infiltration into tumor tissue and recognition of malignant cells are necessary for successful immune elimination. Impaired antitumor activities of T cells and NKs are a major goal of cancer cells. The growth and development of tumors is associated with high alterations in antigenic patterns that lead to attenuated recognition of cancer cells by NK and T cells. At the cancer level, oncomiRs such as miR-22 miR-23a miR-339 tumor cells can affect the activation of immune cells and then control tumor behavior by targeting the expression of the protein like PD-1, NKG2L and ICAM-1, thus preventing the recognition of cancer cells by NK and T cells. In the immune cells oncomiR drive immunosuppressive state by directly or indirectly controlling killing effect of NK cells and affecting the cytotoxicity effect of T cells. The overexpression of oncomiRs in T cells such as miR-23a-3p induces T cell apoptosis through the PD-L1/PD-1 pathway by inhibiting the tumor suppressor PTEN expression and subsequently activates protein kinase B (AKT) (55).

An important notion in the immune system network is that functional alterations in some immune cells may alter the activity of other immune cells. For example, a reduction in the number of dendritic cells (DCs), macrophages or a reduction in key players of the antigen presentation machinery in these cells could lead to ineffective T-cell stimulation and consequently drive a T-cell activation reduction that directly affects T-cell function and polarization (58). Given this, oncomiRs that alter the activity of one class of immune cells can have a major impact on other immune cell types. Therefore, the tumor-derived exosomes released by HCC cells can affect macrophage function, which changes TME polarization and helps the tumor escape immune surveillance. In HCC-derived exosomal miR-146a-5p transferred to macrophages is an essential mediator required for the polarization of macrophages to an M2 phenotype and indirectly drives T-cell exhaustion by M2 TAMs. Exosomal miR-21-5p transferred from macrophages induced CD8+ T-cell exhaustion *via* the miR-21-5p/YOD1/YAP/β-catenin pathway (59, 60) **(** **Figure 2** **)**.

Tumor-derived exosomal oncomiRs modulate immune checkpoint protein expression and can inhibit activated T cells expressing Inhibitor of cysteine peptidase (ICP). This deregulation of ICP expression is associated with the emergence of T-cell exhaustion in many cancers (3). Cancer cells downregulate several miRNAs that negatively target the expression of ICP. For example, the miR-200 family members miR-200a, miR-200b, miR-429 and miR-141, which are known to act as TSs in many cancers (61), are reported to target PD-L1 expression and Zinc Finger E-Box Binding Homeobox 1 (ZEB1) in NSCLC (62). Subsequent expression of these molecules on the surface of cancer cells delivers an inhibitory signal to T cells and NK cells, silencing the host immune response to cancer-specific antigens mediated by these cells (63).Induction of T-cell apoptosis is another immunosuppressive mechanism used by cancer cells. However, a recent study demonstrated that exosomal miR-23a-3p secreted by HCC cells under endoplasmic reticulum stress increased the expression of PD-L1 and inflammatory cytokines in macrophages (69). Several studies have shown that deregulation of PD-L1 expression leads to tumor cells escaping the immune response and promoting cancer aggressiveness (70). Mechanistically, miR-23a-3p inhibits PTEN expression and subsequently activates protein kinase B (AKT), reduces the proportion of CD8+ T cells and induces T-cell apoptosis through the PD-L1/PD-1 pathway (71). Based on miRDB and miRabel databases, the recent Bioinformatic Exploratory Study identified 49 unique miRNA were identified across fourteen different cancers as potentially targeting PD-1 (PDCD1)/PD-L1 (CD274) (72).

One mechanism used by cancer cells to escape the immune system is the deactivation of T cells *via* immune checkpoint inhibitors. Additionally, miR-23a was discovered to be a target of cancer cells in CD8 T cytotoxicity, and miR-23a expression in CD8+ T cytotoxicity inhibited the expression of B lymphocyte-induced maturation protein-1 (BLIMP-1), which is an important regulator required for the development and activation of T cells as well as their recruitment to the site of infection (53, 54, 73). Therefore, in the TME, TGF-β suppresses CD8+ T-cell activity indirectly by controlling miR-23a-mediated posttranscriptional expression of BLIMP-1, and consequently its downstream cytotoxic effectors (73–75).

The primary reasons for immune evasion in cancer are the presence of T-cell dysfunction and excessive suppressor T cells (76). Under prolonged exposure to cancer-specific antigens, through the action of oncomiRs, T cells may evolve to a dysfunctional stage characterized by a weakened antitumor effect (77, 78).

#### 3.2.2 OncomiRs are involved in immune escape in the NK-mediated immune response

In the study of the antitumoral response, most attention is given to T cells, but other competent immune cells, such as NK cells, are also capable of recognizing and eliminating cancer cells. NK cells are considered one of the major types of infiltrating immune cells in many solid tumors and are capable of recognizing nascent tumor cells to prevent tumor development and subsequent metastasis (59, 79). As innate immune effectors, NK cells participate in the early response against tumors, and NK antitumor action is related to interféron gamma (IFN-γ) production and numerous cytotoxic products, such as perforin and granzyme. In TME, the IFN-γ activates both innate and adaptive immunity (80). However, deregulation of miRNA expression in NK cells can affect their function, and overexpression of miR-23a alters the antitumor function of NK cells by directly inhibiting RUNX Family Transcription Factor 1 (RUNX1) (81). MiR-218-5p and miR-24-3p have also been reported to suppress the NK killing effect in CRC by targeting paxillin (PXN) (82) and in lung adenocarcinoma by targeting Serine Hydroxymethyltransferase 1 (SHMT1) (83), respectively. However, several miRNAs were reported to alter the cytotoxic function of NK cells. Overexpression of miR-146a in chronic hepatic carcinoma (HCC) patients impeded NK cells and reduced the production of IFN- γ and TNF-α, which were reversed upon inhibition of miR-146a (84). In this study, treatment with interleukin 10 (IL-10) and Transforming growth factor (TGF-β) induced the expression of miR-146a but was repressed by IL-12, IFN-α and IFN-β. In addition to the direct effect of miRNAs, the deregulation of oncomiR expression in tumor cells can indirectly impact the function of NK cells. The cytotoxicity of these cells was shown to be controlled by several miRNAs. For instance, NK cells express receptors at their surface that are capable of recognizing their target on cancer cells. The NKG2D receptor mediates NK-cell cytotoxic ability and acts as a costimulatory receptor that is involved in T-cell differentiation and expansion (85) **(** **Figure 2** **)**. NK cells recognize malignant cells through the recognition of NKG2L, referred to as MHC I ligand UL16 binding protein 1 (ULBP1-6) and MHC class I-related chain A and B (MICA/B) expressed in cancer cells. All of these molecules are recognized by an NKG2D receptor that is expressed on the cell surface of various cells in the innate and adaptive immune systems (85). In this way, the antitumor response of NK cells is recognized as an important mediator *via* the NKG2D ligand/NKG2D axis. Nonetheless, cancer cells have developed many strategies to downregulate the expression of NKG2DL to escape NKG2D-mediated immune control. One of these strategies is the downregulation of NKG2DL mRNA by the action of miRNAs (86, 87). Several oncomiRs have been reported to downregulate the expression of NKG2DL in multiple cancer types. A group of miRNAs that regulate the expression of MICA and MICB were identified, namely, miR-93, miR-520d, miR-106b, miR-23a and miR-373. These miRNAs are ubiquitously expressed in several cancer tissues and in cancer cell lines (88). Silencing miR-20a, miR-93 and miR-106b expression in glioblastoma cell lines upregulated NKG2DL expression, which led to increased susceptibility of NK cells to mediating lysis (89). In hepatocellular carcinoma, the miR-25-93-106b cluster significantly affected MICA expression, while its inhibition enhanced MICA expression. This change in MICA expression by the miR-25-93-10b cluster led to evasion of the NK response mediated by the NKG2D receptor *in vitro* and in an *in vivo* cell-killing model (90). NKG2DL ULBPs were identified as a strong prognostic marker in human melanoma. ULBP expression has been shown to be controlled by miR-34a, and miR-34c is known as a TS miRNA (91). High levels of these miRNAs lead to upregulated ULBP2 expression, while their inhibition diminishes tumor cell recognition by NK cells. miR-34 expression is controlled by P53, demonstrating miRNA-mediated crosstalk between the immune system and cancer (91). Moreover, miR-140-5p/-409-3p/-433-3p/-650 was reported to regulate the expression of ULBP1, leading to modulation of the NK response in different cancer types (92).

In ovarian cancer, miR-20 was shown to regulate NK cytotoxicity by targeting NKG2DL in cancer cells (93). In breast cancer stem cells, high levels of miR-20 induced a downregulation of MICA and MICB, which consequently decreased the capability of NK cells to mediate cell killing and promote the metastatic ability of cancer cells (79). In addition, NK cells express certain receptors on their surface, such as the chemotactic receptor CX3CR1, which can drive the accumulation of these cells toward peripheral tissues, including tumor sites. CX3CR1 is one such chemotactic receptor expressed on the surface of NK cells. Some oncomiRs target the expression of these proteins, impairing the accumulation of NK cells in the TME. However, increased levels of miR-27a-5p were shown to negatively target the expression of CX3CR1 in NK cells in neuroblastoma, consequently reducing the recruitment of CXCR1 to NK cells in the tumor mass (94, 95). Tumor cell resistance to apoptosis and tumor immune escape are related to miRNAs and NK cells. Overexpression of miR-519a-3p in BC resists apoptosis by interfering with apoptotic signals induced by tumor necrosis factor-related apoptosis-inducing ligand (TRAIL) and Fas Ligand (FasL) but also impairs the anticancer response of NK cells by downregulating NKG2D ULBP2 and MICA, which are important for the appropriate recognition and function of NK cells (96).

#### 3.2.3 OncomiR mediated macrophage polarization

Macrophages are antigen-presenting cells with phagocytic activity and are one of the most numerous immune cells in the TME (97). Macrophages can contribute to tumor development by participating in cancer immune evasion and slowing the antitumor immune response through several mechanisms, such as enhancing angiogenesis and stimulating cancer migration (98, 99). Two classes of macrophages were reported to be involved in cancer development, activated M1 and M2 macrophages, according to their polarization status; M1 macrophages are reported to behave as potential antitumor cells, while M2 macrophages can contribute to tumor progression (100, 101). The regulation of the function and polarization of macrophages by oncomiRs has been extensively reported. High expression of miR-100c maintains the phenotype of TAMs by altering the mammalian target of rapamycin (mTOR) signaling pathway (102). Moreover, interfering with miR-100 expression in TAMs in a mouse BC model inhibited TAM protumoral function and abolished the tumor metastasis and invasion capacities of BC by targeting Interleukin-1 receptor antagonist (IL-1ra) (102). Macrophages can undergo coordinated changes in gene expression due to their high plasticity in response to TME cues, which differentiates them toward a protumoral phenotype with anti-inflammatory and immunosuppressive abilities (103). Therefore, TAMs have a wide ranging impact on tumor initiation.

**Figure 3:**
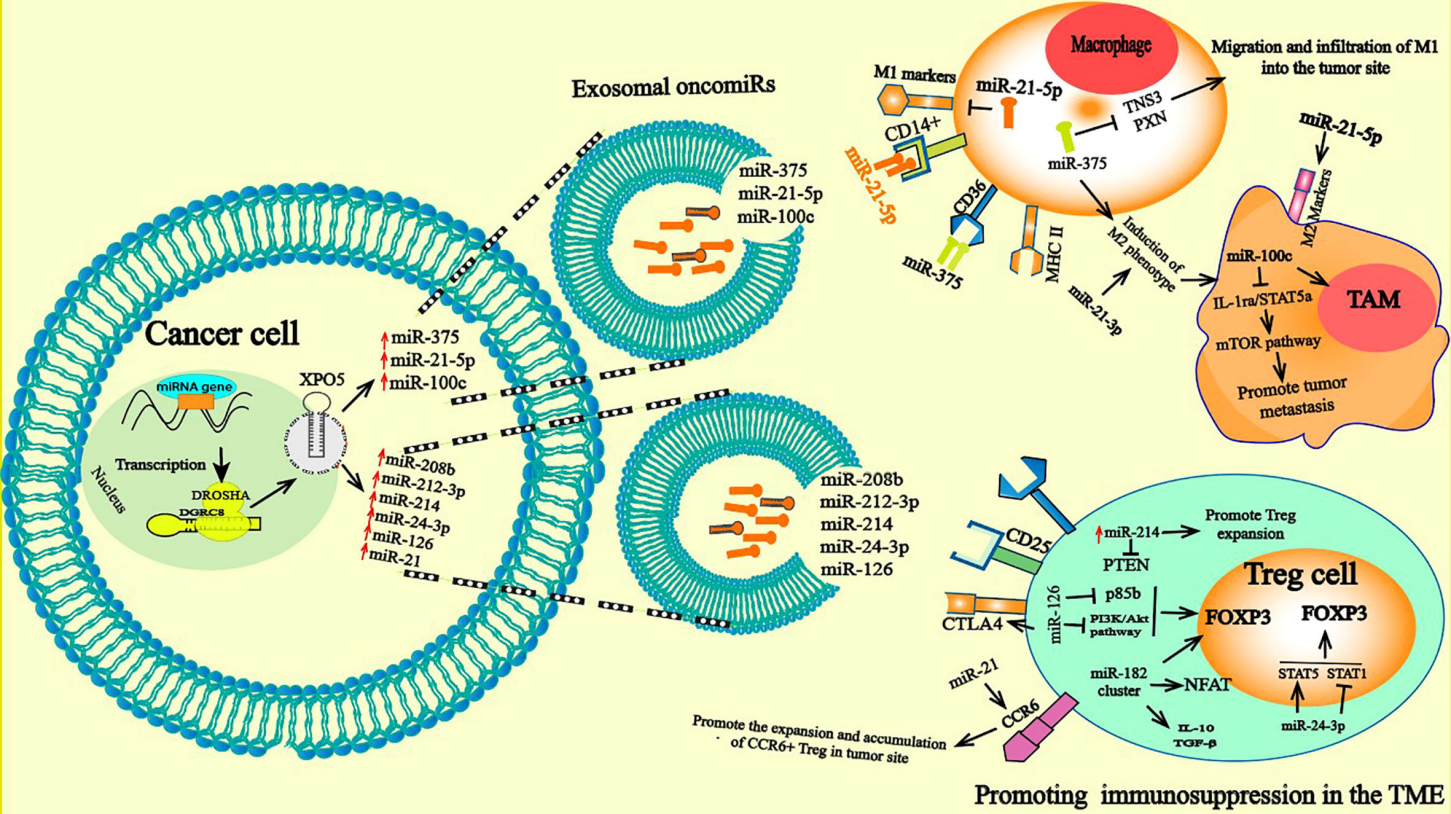
Pro-tumorigenic functions of TAM and Treg cells: miRNAs delivered by tumor derived exosome induce abnormally expressed miRNA in TAM and Treg. In macrophage, exosomal secreted miRNAs can be taken up by the receptor CD36 and CD14. The aberrantly expressed miRNAs in macrophage can directly induce a change in gene expression and consequently polarizes them toward a protumoral phenotype M2 with anti-inflammatory and immunosuppressive proper. A variety of functions of TAM related to cancer progression were descripted; they contribute to tumor cell escape and hinder the antitumor immune mechanism and response through mechanisms such as stimulating angiogenesis, enhancing tumor migration and suppressing antitumor immunity. Treg cells in turn are involved in tumor development and progression by inhibiting antitumor immunity, thereby, facilitating the development of an immunosuppressive TME and promoting cancer progression. The high expression of oncomiRs such onco-miR-182 cluster (miR-96, -182, and -183), miR-126 increased FOXP3, TGF-β, and IL-17 expression and drive polarization of T cells toward the transitional state of IL-17–producing Tregs and elevated frequencies of Tregs leading to further immune suppression by the potent regulatory/suppressive capacities of Tregs.

Secreted exosome containing miR-21-5p, which can be transferred from MSCs to cancer cells and macrophages, promote M2 polarization of macrophages, inducing tumor cell proliferation and immunosuppression in the TME (104). Analogous to this study, it was shown that the EMT activates the M2 phenotype. Under the action of the EMT transcription factor Snail, cancer cells produce miR-21-abundant tumor-derived exosomes. Exosomal miR-21 is absorbed by CD4 monocytes, altering expression of the M1 marker and promoting that of M2 (105). In contrast, overexpression of some TS miRNAs in exosomes, including miR-130 and miR-33, activates M1-related genes (Interferon regulatory factor 5 (*IRF5*), monocyte chemoattractant protein 1 (*MCP1*), *CD80*) and the production of TNF-α, and IL-1β decreases the secretion of TGF-β and IL-10 by these cells. Consequently, this leads to attenuated cancer growth by shifting M1 macrophages to M2 polarization (106). In addition to regulating the polarization of macrophages, oncomiRs have also been shown to regulate other functionalities of TAMs, such as their protumoral effect and their recruitment and migration into the tumor site. Enhanced macrophage migration and infiltration are prominent features of most solid tumors, and play a principal role in tumor progression and metastasis. MiR-375 is released from apoptotic tumor cells as LDL binds a nonexosomal entity that is absorbed by TAMs *via* CD36, which alters the expression of Tensin 3 (TNS3) and PXN, two genes involved in the migration and infiltration of macrophages (103, 107) **(** **Figure 3** **)**. However, several upregulated oncomiRs, including miR-25-3p, miR-130b-3p, and miR-425-5p, encased in the exosome produced by CRC cells were shown to be absorbed by macrophages, inducing activation of the CXCL12/CXCR4 axis by promoting liver metastasis in CRC. TAMs intricately participate in the EMT process of tumor cells. Overexpression of miR-363-3p by the M2 phenotype reportedly promotes the EMT mechanism by regulating CD82 expression in liver cancer cells. Mechanistically, M2 increases the expression of miR-363-3p under the action of the transcription factor Smad2/3 mediated by TGF-β (108).

The communication between TAMs and cancer cells in the TME plays a prominent role in cancer progression (109, 110). Furthermore, TAMs modulate various factors in the TME to facilitate tumor development and progression by promoting cell proliferation and angiogenesis (111). Exosomal miR-501-3p produced by TAMs was reported to contribute to the development of pancreatic ductal adenocarcinoma (PDAC) by inhibiting the TS transforming growth factor beta receptor 3 (TGFβR3) (111). In a recent study, it was shown that exosomes produced by macrophages enhance cancer migration and invasion, exosomal miR-21-5p and miR-155-5p decreased expression levels of the transcriptional activator brahma-related gene 1 (*BRG1*) by directly targeting its sequence (112). Another study showed that hypoxic epithelial ovarian cancer cells (EOCs) reprogram macrophages to a TAM phenotype and trigger macrophage recruitment. Exosomal miR-223 derived from TAMs under hypoxia enhanced the malignant phenotype of EOC cells, accompanied by enhanced anticancer drug resistance in EOC cells by activating the PTEN-PI3K/AKT pathway (113). This explains the manner in which immune cells participate in cancer progression and unveils new insights regarding the interactions between tumor cells and macrophages. In summary, miRNAs are pivotal in the control of tumor development by TAMs. Polarization of macrophages occurs during tumor growth depending on the inflammatory context and is due to the simultaneous influence of both anti- and protumorigenic signals. In this context, macrophage differentiation depends on the inflammatory status of the TME (114). TAMs, the most prominent cells in the TME, play a crucial role in cancer progression, dependent on signaling in the TME that reprograms them into a protumoral phenotype, M2, or an antitumor phenotype, M1.

#### 3.2.4 T regulatory cells and the antitumor immune response

Induced Tregs are generated by on-site polarization of CD4+ cells, defined as CD4+ T cells with high expression of CD25 (115) and Foxp3. Foxp3 is a member of the Forkhead/winged helix family and is a pivotal lineage-specific transcriptional regulator of Tregs (116). Treg infiltration into the tumor site constitutes a strong barrier against effective antitumor immune responses and largely contributes to the development of an immunosuppressive TME that promotes tumor growth. In recent studies, the accumulation of FoxP3+ Tregs within the TME is clearly associated with a worse prognosis in ovarian and pancreatic ductal adenocarcinomas (117, 118). However, miRNAs play a prominent role in the regulation of Treg development and functions (119–122). For example, high expression of miR-155 is maintained by expression of the transcription factor FOXP3, which binds to the promoter region of the *BIC* gene, which hosts miR-155 and encodes the primary RNA precursor of miR-155 (123). Reduced expression of miR-155 impairs the proliferative potential of Tregs, inducing significantly decreased numbers of thymic and splenic Treg cells. The reduction in miR-155 expression was shown to be caused by attenuated IL-2 signaling mediated by high expression levels of suppressor of cytokine signaling 1 (SOCS1), which is a direct target of miR-155 and a negative regulator of the IL-2 receptor (123). High expression of the oncomiR-182 cluster (miR-96, -182, and -183) in patients with BC increases the expression of Foxp3, TGF-β, and IL-17, inducing polarization of T cells into the transitional state of IL-17–producing Tregs and elevates numbers of Tregs in BC (124). It has been reported that overexpression of miR-182 results in a dramatic reduction in the gene expression of *FOXO1*, the *TCR/CD3* complex, *NFAT*, and *IL-2/IL-2RA* on T cells by targeting signaling pathways (124). OncomiRs can regulate the differentiation and suppressive function of Tregs. However, overexpression of miR-126 promotes the production of Tregs and stimulates the function of these cells by suppressing p85b expression and altering activation of the PI3K/Akt pathway. It has been noted that silencing miR-126 alters Foxp3 expression on Treg cells, which is followed by reduced expression of CTLA-4, Tumor necrosis factor receptor superfamily member 1 (GITR), IL-10 and TGF- β, decreasing the induction of Tregs, impairing their suppressive function and disrupting the antitumor activity of CD8+ T cells (125). OncomiRs also regulate other functions of Tregs in tumor immune cells. In CRC, the increased expression of miR-208b in exosomes derived from CRC cells was recently reported to play pivotal roles in tumorigenesis by stimulating Treg proliferation *via* targeting PDCD4, thereby repressing the anticancer immune response, which in turn accelerates tumor progression and reduces oxaliplatin chemotherapy sensitivity (126). Exosomal miR-214 derived from tumor cells and delivered into peripheral CD4+ T cells in a mouse model downregulated PTEN, promoted Treg expansion and enhanced immune suppression (28). In addition, miR-24-3p-containing exosome in NSLC are reported to induce an elevated frequency of Tregs by inducing Signal Transducer and Activator of Transcription 5 (STAT5) and inhibiting STAT1 and pREK (127). Increased levels of miR-21 in tumor tissues from a murine breast cancer model reportedly stimulate the proliferation of CCR6+ Tregs. Notably, inhibition of miR-21 expression significantly decreased the proliferation of C-C Motif Chemokine Receptor 6 (CCR6+) Tregs, altered the recruitment of CCR6+ Tregs into the tumor site and effectively endowed CD8+ T cells with an anticancer function by altering the PTEN/Akt axis (128) **(** **Figure 3** **).**

OncomiRs also affect the balance of Th17/Treg cells in cancer. It has been reported that the imbalance between Treg and Th17 cells controls the immune response, which has been characterized in many cancer types. Resting naïve CD4+ cells proliferate and differentiate into different CD4+ subpopulations depending on the microenvironment-promoting signals, including Th1, Th2, Th17 and Treg cells. Among these cells, Tregs mediate immunosuppression by secreting IL10 (129), while Th17 cells releasing IL17 mediate inflammatory activity and strengthen the defense response (130, 131). In a normal environment, Th17 cells and Tregs exist in equilibrium. Th17/Treg imbalance was reported to be mediated by PD-1/PD-L1 and partially affected by high expression of miR-21 in gastric cancer (132). Moreover, miR-155 was shown to induce Th17/Treg imbalance by regulating the progression of cervical cancer (CC) by inhibiting SOCS1 expression (133). Furthermore, TAM-released exosomes carrying miR-29 and miR-21-5p can be absorbed by CD4+ cells and induce an imbalance between Treg/Th17 cells by directly inhibiting STAT3 in epithelial ovarian cancer (109).

### 3.3 The existence of oncomiRs in exosomes implies their potential value as biomarkers

Upregulation of oncomiRs in tumor cells is correlated with the progression of many cancer types and can be used as a potential biomarker for the detection of tumor initiation and cancer progression. The use of miRNAs present in exosome as biomarkers for diagnosis and prognosis has many advantages: they are resistant to RNases, are more stable than mRNAs, can be easily detected, are widely available in all body fluids using noninvasive methods and can be constantly monitored (134). Exosomal miRNAs in the biological fluids of patients with different cancer types can be used as a diagnostic tool for tumors driving immunosuppressive responses. Exosomes can contain a tumor-specific oncomiR, which could correlate with tumor development, poor outcome and interference with antitumor immunotherapies. Screening of exosomal miRNAs from body fluids allows us to determine the initiation, progression and aggressiveness of tumors. Some specific exosome containing oncomiRs have high diagnostic value in cancer. For example, some oncomiRs are overexpressed in the plasma-derived exosome of HCC, including miR-21-5p, miR-10b-5p, miR-221-3p and miR-223-3p, and their high expression represents an early diagnostic molecular marker for HCC (135). Moreover, miR-1246 is used in early cancer diagnosis, indicating poor prognosis and distant metastasis in breast cancer and prostate cancer (136–138). In addition, overexpression of serum exosomal miR-10b-5p was found to be related to the development and progression of HCC and significantly associated with poor disease-free survival, making it a potential biomarker for early-stage HCC (48). Moreover a recent study in HCC shows that the down expression of miRNA-29c as TS miRNAs, associated with the overexpression of miRNA-21 and miRNA-155 as oncomiRs, were potential plasma biomarkers predictive of clinical progression and survival (139). Exosomal miR-126a was reported to induce metastatic activity in lung cancer (140). In addition, increased exosomal miRNA expression was shown to correlate with tumor staging. Exosomal miR-224 was reported to be a tumor promoter related to the development of HCC and has potential biomarker value in the diagnosis of advanced tumor stages in HCC (141). Similarly, another study demonstrated that serum exosomal miR-665 was correlated with tumor size and staging (142). In HCC, high expression levels of serum exosomal miR-92b stimulate the invasive activity of liver cancer cell lines. This circulating exosomal miR-92b predicts the risk of posttransplant HCC recurrence. These results identified hepatoma-derived exosomal miR-92b as a prognostic biomarker for the early diagnosis of posttransplant HCC recurrence (143). Recently, preliminary data on the association of miR-10a-5p, miR-19b-3p, miR-215-5p, and miR-18a-5p significant upregulation in gastric cancer patient’s exosomes is also suggested as a new potential early diagnostic non-invasive biomarker. MiR-1290 in EVs derived from gastric cancer cells promote T cell suppression by up-regulating PD-L1 *via* the Grhl2/ZEB1 pathway (144). Functional screening designed to quantify expression levels of exosomal miRNAs in plasma is less noxious to the human body, and the miRNAs are relatively stable and easy to collect, which is helpful for the early prediction and prognosis of cancer. However, the study of exosomal miRNAs remains a challenging task. Thus far, the most widely used exosome isolation and characterization techniques are ultracentrifugation or commercial kits, followed by the use of a nanosight microscope to quantify and detect their diameter size (30-100 nm) and flow cytometry or western blotting to determine surface biomarkers (CD81, CD82 CD9, and CD63). Additionally, this protocol requires sophisticated equipment, high sample volumes and is time intensive. However, other appropriate methods of exosome separation have been explored, such as ultrafiltration, which is more rapid than ultracentrifugation and does not require advanced equipment. Moreover, liquid chromatography has been used to purify exosomes with high efficiency and high purity but requires advanced technology. In conclusion, the approach used to extract exosomes has not been rigorously verified or streamlined. Therefore, the most commonly used method is to combine different approaches. After extraction and characterization of the exosomes, RNA sequencing or qRT–PCR is used to quantify exosomal-encapsulated miRNAs. Furthermore, the detection of exosomal miRNAs is often hindered by time-consuming procedures, such as RNA extraction, cDNA conversion and target amplification, as well as the low levels of miRNAs in exosome, further complicated by delicate noncoding RNA degradation over time after extraction. Other quantitative methods have also been used to better quantify miRNAs encased in exosome. An alternative *in situ* method for the detection of miRNAs encased in exosome without isolation and purification of exosomes from plasma samples was used to detect miRNAs in exosome (145). Au nanoflares (gold nanoparticles functionalized with fluorophore-labeled DNA sequences) are synthetized DNA probes (145) that can directly enter exosomes to generate fluorescent signals that can quantitatively evaluate the level of miRNAs by specifically targeting miRNAs encased in exosome with a high level of sensitivity and specificity (146, 147). This method was used to evaluate miR-1246 in terms of specificity, accuracy and efficiency as a potential biomarker for the diagnosis of BC (136). However, these methods cannot detect low levels of miRNAs or differentiate exosomal miRNAs from total circulating miRNAs, restricting their use in clinical adaptation (148). Likewise, other methods have been reported for detecting miRNAs in exosomes, such as thermophoresis which based primarily on the directed migration of exosomes along a temperature gradient (149). A thermophoretic biosensor implemented with nanoflares allows an increase in the fluorescence signal upon its binding to miRNAs, leading to direct and quantitative levels of exosomal miRNAs with high sensitivity. Exosomal miR-375, a biomarker for the detection of estrogen receptor-positive BC patients during early stages (stages I and II), exhibited an accuracy of 85% (148). Developing additional methods to accelerate the detection of effective, specific, sensitive exosomal miRNA biomarkers is a promising avenue for the early detection of cancer.

### 3.4 Therapeutic methods targeting exosomal miRNAs

Owing to the ability of miRNAs to act as oncomiRs or TS miRNAs, miRNAs have become potential candidate in cancer therapeutic strategies. So far, the most miRNAs utilized in clinical trial are used as biomarkers in diagnosis and prognosis. However, some miRNAs have rich the clinical trials in the treatment of cancer and hepatitis, the most known are the TS miRNAs miR-34 for the treatment of cancer and miR-122 for the treatment of hepatitis, but no-one as oncomiRs. Nevertheless, oncogenic miRNA inhibition was shown to exert significant antitumor activities in cancer, slowing its progression. Several clinical studies using mice model and antagomiRs encapsulated in lipid nanoparticles have shown their great potential as therapeutic agents in the inhibition of cancer growth. In a Triple-Negative Breast Cancer (TNBC) mice model, treatment by anti-miR-21 encased in nanoparticles showed specific uptake to breast cancer stem cells (BCSCs) and TNBC cells and high efficacy for tumor growth inhibition (150). Similarly, treatment with nanoparticles containing, anti-miR-210 showed encouraging results in a mouse model of pancreatic cancer, anti-miR-375 and miR-141 in a prostate carcinoma xenograft model, and anti-miR-150 and anti-miR-638 in advanced melanoma, leading to profound tumor growth inhibition (151). In future approaches using miRNA-based therapies, the administration of miRNA inhibitors, such as antisense oligonucleotides that encode a complimentary sequence to mature miRNAs known as antagomiRs, could inhibit the effect of miRNAs in cancer and block its growth. The use of antagomiRs to inactivate miRNAs in cancer has indeed recently emerged as a promising approach. Notably, antagomiRs are specifically designed to be more stable and less toxic. Furthermore, a wide number of active molecules isolated from natural herbs have been tested in the clinic to treat several tumors. D Rhamnose β-hederin (DRβ-H), a triterpenoid saponin, was reported to inhibit BC cell proliferation by remolding the TME, reducing exosome secretion. DRβ-H inhibits cell growth and induces apoptosis of BC cells (MCF-7/S) by regulating exosome secretion that transports miR-130a and miR-425, which was also decreased after treatment with DRβ-H (152, 153). DRb-H has been shown to reverse docetaxel resistance by inhibiting exosome secretion. The authors demonstrated that DRβ-H decreases expression of the most highly increased oncomiRs, such miR-16, miR-23a, miR-24, and miR-27a, carried by exosomes that are responsible for chemoresistance (153). Moreover, shikonin is another bioactive component of herbal plants and is used as a potential chemopreventive agent; it shows potent antitumor effects for the treatment of many cancers. Shikonin was reported to suppress the migration and invasion of BC cells and glioblastoma cells (154, 155). Shikonin suppresses MCF-7 growth by modulating tumor-released exosomes accompanied by downregulation of miR-128 in BC (156). Moreover, shikonin alters preadipocyte-derived exosomes that promote tumor development by disrupting the miR-140/SOX9 axis during early-stage BC (157). Docosahexaenoic acid (DHA) is another natural molecule with antitumor and antiangiogenic effects that is reported to regulate exosome release to the TME. The authors reported that treatment with DHA increased the expression of some TS miRNAs, such as miR-23b and miR-320b, in exosomes derived from the BC cell lines MDA-MB-231, ZR751 and BT20. Delivery of these miRNAs into endothelial cells by exosomes secreted from BC cells reduced the expression of their proangiogenic target genes (PLAU, AMOTL1, NRP1 and ETS2) and inhibited angiogenic capability by endothelial cells (158). Multiple lines of evidence have indicated that exosomes have significant potential as important carriers and can be used to deliver TS miRNAs or antagomiRs to change the biological behavior of cancer cells. The use of engineered exosomes to deliver specific miRNAs in cancer therapies has been an exponentially increasing field of study in recent years. Using engineered exosomes to systematically co-deliver the anticancer drug 5-FU and a miR-21 inhibitor (miR-21i) to Her2-expressing tumor cells, targeted colon cells underwent reversed tumor resistance with promotion of cytotoxicity in 5-FU-resistant tumor cells (159). Previously, Her2 affinity was fused to the extracellular N-terminus of human LAMP2 to efficiently target HCT-1165FR colon cancer cells. Elucidating the immunosuppressive profile of the exosomal miRNA mechanism in the TME in the context of immune dysfunction in cancer progression and poor outcome is crucial for novel and efficient therapies. For example, the dysfunction of NK cell alters expression of the NKG2D receptor and has been leveraged for therapeutic development using human exosomes harboring NKG2D ligands on their surface, leading to direct activation of NK cells and resulting in the killing of transformed cells. These findings highlight the ability of exosome-based vaccines to reprogram the antitumor capacity of NK cells (160).

## 4 Conclusion

Currently, it has become obvious that miRNAs are widely involved in the interactions between cancer and immune cells in the TME. MiRNAs with oncogenic characteristics regulate cancer progression through several mechanisms, including inhibition of TS genes, suppression of immune cell antitumoral function, and especially modulation of suppressive immune cell function, such as TAMs and Tregs, in favor of cancer growth. Exosomal oncomiRs, which are actively produced by cancer cells, play a crucial role in the TME to support tumor progression and invasion. Several studies have provided mechanistic evidence that exosomal miRNAs absorbed by target cells can modulate gene expression patterns and thereby indirectly promote the development and invasion of cancer cells. The immune system is an important component of the TME. Several studies have shown that immune cells recognize cancer cells and could potentially eliminate them. Nevertheless, cancer cells develop numerous escape and resistance mechanisms; they can alter the expression of tumor antigens on their surface and increase the level of immune checkpoint proteins to modulate immune gene expression to disrupt the recognition of cancer cells by the immune system, contributing to immune evasion. Although our current understanding of the impact of miRNA expression on cancer progression is still in its early stage, the major role of oncomiR mediated interactions between immune and cancer cells opens the door to the development of therapeutic strategies targeting oncomiRs. In this review, we summarized the most common roles of oncomiRs in the development of cancer, focusing on recent advances in their role in regulating critical signaling pathways of cancer cells, and we discussed the potential application of exosomal oncomiRs as novel cancer-related biomarkers as well as their application in the treatment of cancer.

## Author contributions

The first author wrote the first draft. All authors contribute to editing and improving the manuscript and checked the literature for accuracy of references. All authors contributed to the article and approved the submitted version.

## Conflict of interest

The authors declare that the research was conducted in the absence of any commercial or financial relationships that could be construed as a potential conflict of interest.

## Publisher’s note

All claims expressed in this article are solely those of the authors and do not necessarily represent those of their affiliated organizations, or those of the publisher, the editors and the reviewers. Any product that may be evaluated in this article, or claim that may be made by its manufacturer, is not guaranteed or endorsed by the publisher.
